# A Canopy Density Model for Planar Orchard Target Detection Based on Ultrasonic Sensors

**DOI:** 10.3390/s17010031

**Published:** 2016-12-24

**Authors:** Hanzhe Li, Changyuan Zhai, Paul Weckler, Ning Wang, Shuo Yang, Bo Zhang

**Affiliations:** 1 College of Mechanical and Electronic Engineering, Northwest A&F University, Yangling 712100, China; lihanzhe187@163.com (H.L.); yangshuosjz@163.com (S.Y.); zhangbo_609@163.com (B.Z.); 2 Department of Biosystems and Agricultural Engineering, Oklahoma State University, Stillwater, OK 75078, USA; paul.weckler@okstate.edu (P.W.); ning.wang@okstate.edu (N.W.)

**Keywords:** precision spray, target detection, canopy density model, ultrasonic sensor, orthogonal regression central composite experiment

## Abstract

Orchard target-oriented variable rate spraying is an effective method to reduce pesticide drift and excessive residues. To accomplish this task, the orchard targets’ characteristic information is needed to control liquid flow rate and airflow rate. One of the most important characteristics is the canopy density. In order to establish the canopy density model for a planar orchard target which is indispensable for canopy density calculation, a target density detection testing system was developed based on an ultrasonic sensor. A time-domain energy analysis method was employed to analyze the ultrasonic signal. Orthogonal regression central composite experiments were designed and conducted using man-made canopies of known density with three or four layers of leaves. Two model equations were obtained, of which the model for the canopies with four layers was found to be the most reliable. A verification test was conducted with different layers at the same density values and detecting distances. The test results showed that the relative errors of model density values and actual values of five, four, three and two layers of leaves were acceptable, while the maximum relative errors were 17.68%, 25.64%, 21.33% and 29.92%, respectively. It also suggested the model equation with four layers had a good applicability with different layers which increased with adjacent layers.

## 1. Introduction

Prevention of insects and diseases of crops is a crucial factor in orchard management. In conventional agriculture chemical spray application is still the main way to insure high yields at a low cost. However, excessive pesticide application results in residues on fruits and soil, which pollute the environment and threaten the safety of agricultural products. Precision target-oriented variable spraying is an effective method to reduce pesticide residue. To achieve this goal, real-time acquisition of the orchard targets’ characteristic information is the key.

The characteristic information of the orchard target includes the tree’s diameter, volume, Leaf Area Index (LAI), canopy density, etc. Many researchers have applied ultrasonic techniques, digital photographic techniques, optical sensors, high-resolution radar images, high-resolution X-ray computed tomography, stereo vision and LIDAR (light detection and ranging) sensors for target characteristic information acquisition [1,2,3,4]. Crop management plans including spraying, irrigation and fertilization have benefited from the application of targets’ characteristic information [1,2]. Especially in air-assisted variable-rate spraying, a controller adjusts the spraying parameters which include pesticide flow rate and airflow rate based on targets’ characteristic information to improve the performance of spraying [3,4]. Light interception and aerial photogrammetry have been used to measure the shape and size of trees, which were sufficient for plant protection [5,6]. The computerized spraying control system with ultrasonic measurement arrays and GPS (global positioning system) or DGPS (differential global positioning system), can automatically adjust pesticide flow rate according to real-time sensing, monitoring, calculation, storage and mapping of tree canopy volume and height [7,8,9,10]. The distance from a sprayer to orchard targets at different heights can be measured using several ultrasonic ranging sensors, and the trees’ volumes can be estimated based on a neural network algorithm [11,12,13]. But the sound cone determination, angle errors, crosstalk errors and field measurements were affected by surroundings [14]. LIDAR has been widely used in measuring 3D (three-dimensional) structural characteristics of trees including the target’s height, width, volume, leaf area index and leaf area density [15]. The unstructured point cloud is obtained from LIDAR scanning, and then the computer processes the point cloud data and rebuilds the 3D digital model of the target. This method allows fast and nondestructive measurement of a target’s parameters and also has a high correlation with actual measurement [16,17,18,19]. LAI is an important indicator in determining the growth status of plants. Some researchers also use the digital photographic techniques to estimate LAI. Compared to the other methods this estimating method observably expand the spatial area and frequency of analysis [20,21,22]. The LIDAR also has been used in drift detection and crop discrimination to guide spraying [23,24].

Integrating many different types of target characteristic information, Walklate compared spray volume deposition based on different models including a vertical wall area model, cylindrical wall area model, tree row volume model, tree area index model, tree area density model and light interception flux model. The result suggested that the tree area density is one of the most important parameters of a single tree target [25]. Ultrasonic techniques, digital photographic techniques and LIDAR have been used in detecting orchard target canopy density, but they still lack quantitative/parametric equations. Palleja estimated canopy density using ultrasonic envelope signals [26]. The results showed that ultrasound’s wave intensity can be used as an indicator of canopy density, however it could only reflect the change of density and lacks a quantitative relationship between density and ultrasound’s wave intensity. It could not provide the control basis for real-time mathematical equations in variable spraying. This paper aims to explore the quantitative relationship between ultrasounds’ wave intensity energy and canopy density, and establish the orchard target canopy density model.

## 2. Materials and Methods 

### 2.1. Target Canopy Density Detection Method

In order to measure the intensity of ultrasonic echo, an ultrasonic sensor XL-MaxSonar MB7092 (MaxBotix Inc., Brainerd, MN, USA) was used. The sensor operated on 3.0 V–5.5 V with five functional pins. It could output analog voltage of range measurement at pin3, and output the analog voltage envelope of the acoustic waveform at pin2. The other three pins were controlling pins. The echo analog voltage of ultrasonic sensor was recorded from pin2 to analyze echo intensity. Echo intensity was influenced not only by the target distance and the target spatial dimension, but also by the canopy density. 

Time-domain energy analysis is a common method of signal analysis. The time-domain energy calculation method is as follows:

(1) $$E=\int_{-\infty }^{+\infty }x^{2}\left(t\right)dt$$

(2) $$E=\sum_{k=0}^{n}x^{2}\left(k\right)$$

where *E* is the energy of signal, *x*(*t*) is the analog signal and *x*(*k*) is the sequence of digital signal.

**Figure 1:**
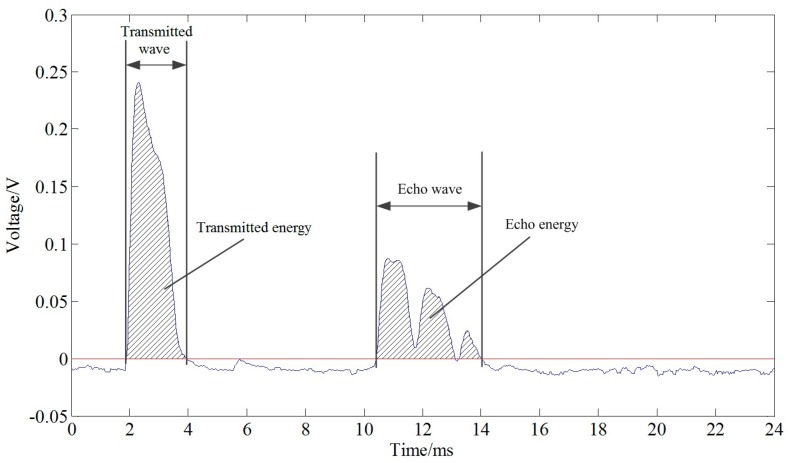
Ultrasonic transmitted wave and echo wave.

The output signal voltage representing the ultrasonic wave is shown in Figure 1. This graph displays the transmitted wave and echo wave. The ultrasonic energy was analyzed based on these waves.

The ultrasonic transmitted energy and echo energy were calculated using MATLAB software (MathWorks, Natick, MA, USA). The ultrasonic signal was recorded by an oscilloscope and a computer. The signals of the transmitted and echo waves should not be negative in theory. The negative data was treated as zero. Then the signal was smoothed using the smooth function in MATLAB. The transmitted energy and echo energy were calculated after the signal processing.

### 2.2. Target Density Detection System

**Figure 2:**
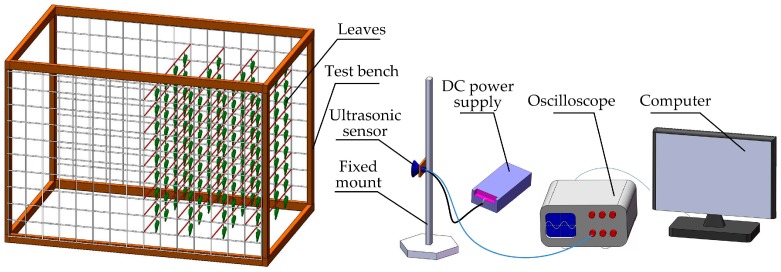
Diagram of target density detection system.

The target density detection system included a test bench, an ultrasonic sensor, a fixed mount, a DC power supply, an oscilloscope and a computer. The detection system was developed as shown in Figure 2. It provided a controllable test environment in which density and detecting distance could be accurately adjusted.

The test bench consists of a wooden frame, fishing lines and wire fencing. The size of the wooden frame (length × width × height) was 150 cm × 103 cm × 103 cm. The fishing line with a diameter of 0.234 mm had almost no effect on ultrasonic echo waves. The wire fencing was fixed to two sides of the wooden frame to attach fishing lines. The grid size of the wire fencing was 1 cm × 1 cm. Fishing line crossed the wire fencing grid in the same plane to constitute a layer, the spacing of fishing line was 5 cm. In each layer there were nine rows of two fishing lines. The leaves could be clamped on each row using clips. The spacing of layers was 20 cm, therefore the volume of each layer was 0.188 m^3^. The leaves of the Chinese glossy privet (*Ligustrum lucidum*) were chosen for the experiment. The weight of each leaf was between 1.0 and 2.0 g, while the size of the leaf was about 10 cm × 6 cm. Under such conditions, the maximum weight of leaves that could be arranged in each layer was 212 g, while the maximum density of each layer was 1127.66 g/m^3^. The minimum density was set as 112.77 g/m^3^, which was 10% of the maximum density. In the test bench several layers of leaves could be combined to simulate canopies with different thicknesses. The density of each layer was the same. The leaves were evenly fixed in each row with interspersed arrangements in adjacent rows. In the adjacent layers, the arrangements were interspersed as well. 

The DC power supply was S-25-5 5V DC power supply (Weiming Power, Qidong, China), whose actual voltage output was 5.69 V. The oscilloscope used was a RIGOL DS1062E-EDU (Beijing RIGOL Technology Co. Ltd., Beijing, China), which recorded the waveform from an ultrasonic sensor. The Ultrascope for DS1000E Series software was used to read the waveform of oscilloscope on the screen. This software could save the waveform as a BMP picture and an Excel file to a computer through an RS-232 to USB converter. 

### 2.3. Experiment for the Relationship between the Ultrasonic Energy and the Power Supply Voltage

During practice use of the ultrasonic sensor, it was found that the ultrasonic energy would change with its power supply voltage. An experiment was designed to establish the relationship between the ultrasonic energy and the power supply voltage. The sensor was powered by an MPS-3003L-3 laboratory power supply (Matrix Technology Inc., Shenzhen, China), whose voltage range was 0–30 V with the regulation precision of 0.1 V. Since the ultrasonic sensor accepted a power of 3.0–5.5 V，the voltage of the power supply was set between 3.0 and 6.0 V with a current of 200 mA. A smooth solid wall was used as a test target, which was 1.0 m away from the surface of the sensor. An oscilloscope and a computer with a DS1000E Series software Ultrascope recorded the waveform when the power supply was set from 3.0 V to 6.0 V with increments of 0.1 V. In each treatment, the waveform data was recorded three times. The averages of transmitted energy and echo energy were calculated using MATLAB to analyze the relationship between the ultrasonic energy and the supply voltage.

It was meaningful to analyze the echo energy under a unified transmitted energy, but it was difficult to keep the supply voltage constant. Thus normalization of transmitted energy through a mathematical method was determined. The fitting equation between the correction coefficient and supply voltage was obtained using the CFTool in MATLAB.

### 2.4. Experiment for Beam Width of Ultrasonic Sensor

**Figure 3:**
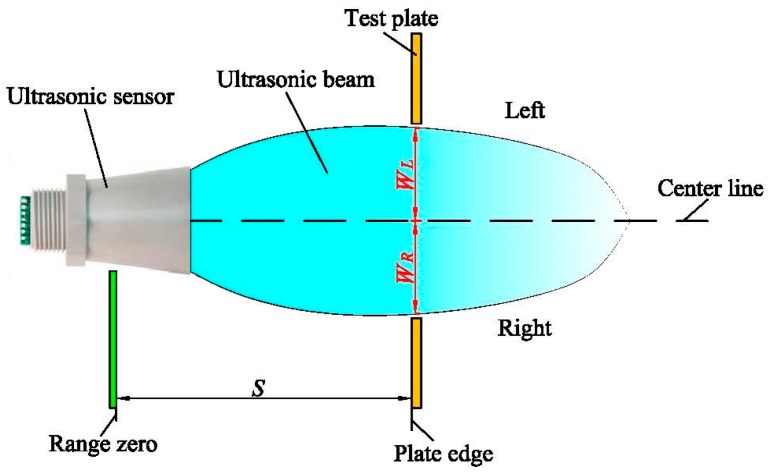
Diagram of measuring method for the beam width of an ultrasonic sensor.

The beam width of the ultrasonic sensor is an important parameter which determines the detecting range. The diagram of the measuring method to obtain the beam width at different detecting distances is shown in Figure 3, where *S* is the detecting distance between the ultrasonic sensor and the test plate edge; *W*_R_ is the distance between the center line and the right test plate; and *W*_L_ is the distance between the center line and the left test plate. The value of *S* was calculated in the orthogonal regression central composite experiment (will be mentioned in Section 2.5). 

In the measurement experiments, the ultrasonic sensor was placed in an empty space where the sensor couldn’t receive any echoes. The oscilloscope read the waveform output of the ultrasonic sensor in real-time. A test plate was moved slowly from right (or left), to the center line until the ultrasonic sensor received echoes. Then, the distance *W_R_* or *W_L_* was manually measured between the test plate and the center line. Each measurement was conducted 3 times. The final value of *W_R_* or *W_L_* was the average of the 3 repetitions. The beam width was the sum of *W_R_* and *W_L_* which must be measured at the same detecting distance *S*.

### 2.5. Orthogonal Regression Central Composite Experimental Design

A central composite design is the most commonly used response surface designed experiment. Central composite designs are a factorial or fractional factorial design with center points, augmented with a group of axial points (also called star points) that allow estimation of curvature. The orthogonal regression central composite experimental design is an effective method to obtain mathematical relationships between factors and variables [27,28]. Only the representative test points are chosen from the comprehensive full-scale tests based on orthogonality, which makes this method more efficient by reducing test times. Canopy density models were designed to be obtained based on orthogonal regression central composite experiments. The factors were the density and the distance, while the result was the echo energy of the ultrasonic sensor. The parameter *γ* which was used to determine factors levels, was calculated by the following equations:

(3) $$m_{c}-\frac{m_{c}^{2}}{n}-\frac{4m_{c}}{n}\gamma^{2}-\frac{4}{n}\gamma^{4}=0,\gamma =\sqrt{-2^{p-1}+{\left(2^{p}+2p+m_{0}\right)}^{\frac{1}{2}}\times 2^{\frac{p}{2}-1}}$$

where *p* is the number of factors; *m_c_* is the number of orthogonal tests; *m*_0_ is the number of the zero level repeat tests; *n* is the number of the total tests; *γ* is star test point parameter; and *m*_0_ is the number of zero level repeat tests. In these orthogonal regression experiments, parameter *m*_0_ was set as: *m*_0_ = 3. The values of the other parameters were: *p* = 2, *m_c_* = 4, *n* = 11, *γ* = 1.15:

(4) z0j=(zlj+zuj)2,Δj=(zuj−zlj)2r,xj=(zj−z0j)Δj

where: *Z_lj_*, *Z_uj_* and *Z*_0*j*_ are the lower level, upper level and zero level of the factor j respectively; *Δ_j_* is the range radius; *Z_j_* is the value of factor *j*; and *x_j_* is the factor level code. The factor levels coding is shown in Table 1.

**Figure 4:**
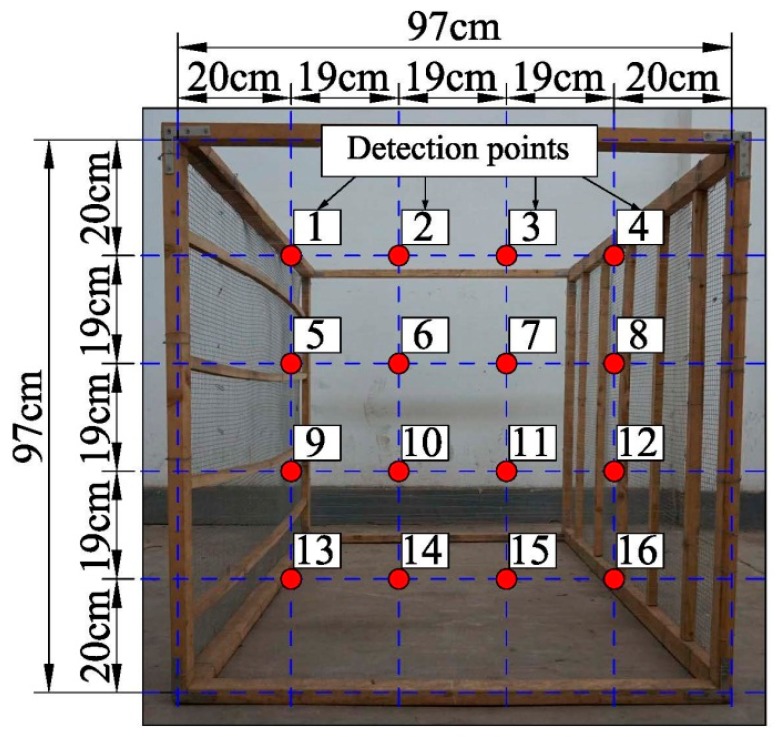
Distribution diagram of detection points.

In orthogonal experiments, the detection points distributed on the test bench were set due to the results of the beam width experiment (will be mentioned in Section 3.2). The distribution diagram of detection points is shown in Figure 4.

According to the results of the beam width experiments, the maximum of the beam width was 20 cm. The detection points were set 20 cm inside from the test bench boundary, while the spacing of adjacent detection points was 19 cm. At each of the 16 detection points, an average of echo energy was obtained by three replicated measurements. In the 16 echo energies, the three maximum and the three minimum ones were removed, and the 10 left were averaged to generate the final echo energy data to establish orthogonal regression equations. The regression equations were calculated by the following computational process:

(5) $$y=b_{0}+\sum_{j=1}^{p}b_{j}x_{j}+\sum_{j<k}b_{jk}x_{j}x_{k}+\sum_{j=1}^{p}b_{jj}x_{j}^{\prime }$$

where *b* is the coefficient of regression equation; and *y* is the echo energy calculated by regression equation. The calculation of coefficients was omitted, but the detailed calculation process can be obtained from [27,28].

For the sake of confirming the reliability of the equation established, the regression equation and its parameters were hypothesis tested using the following expressions:

(6) Fj=SjfjSefe;F=STfTSRfR;

where *S_j_* are the sums of partial regression squares; *f_j_* is the degree of freedom of *S_j_*; *S_e_* was the sum of error squares within repeat test group; *f_e_* was the degree of freedom of *S_e_*; *S_T_* was the sum of regression squares; *f_T_* was the degree of freedom of *S_T_*; *S_R_* was the sum of residual squares; *f_R_* was the degree of freedom of *S_R_*; *F_j_* was the F distribution statistic of parameter *j*; and *F* was the F distribution statistic of the regression equation. The significant coefficients will be selected to build the regression equations based on the *F*-test.

In case of the repeated measurement data, the model can be evaluated by test for lack of fit. The test for lack of fit of canopy density model was calculated by the following equation:

(7) Flf=SlfflfSefe

where *S_lf_* is the sum of lack of fit squares; *f_lf_* is the degrees of freedom of *S_lf_*; and *F_lf_* is the F distribution statistic used in the test for lack of fit.

**Figure 5:**
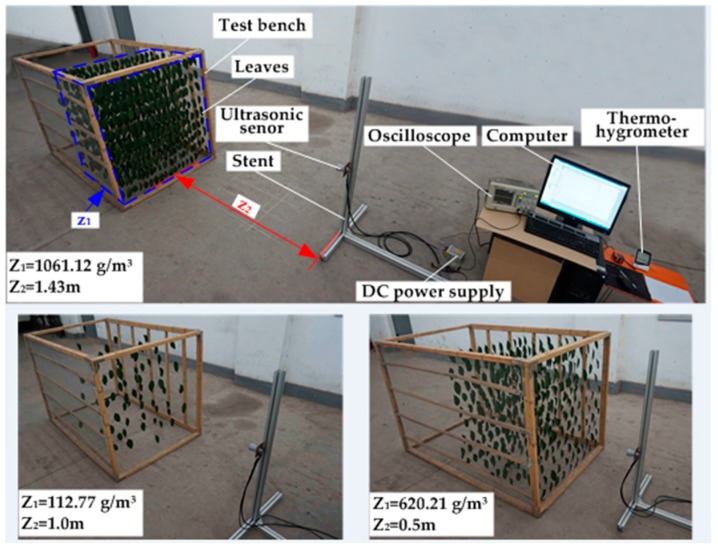
Canopy model experiments performance with four layers of leaves.

In order to establish a canopy density model, orthogonal experiments were conducted with three and four layers of leaves, and two canopy density models were obtained based on three layers and four layers. A verification experiment was conducted to select a better model. The experiments for establishing canopy density models were performed indoors. Canopy model experiments with four layers are shown in Figure 5. Each experimental datapoint was recorded three times, and the average data was used as the result. The final result was a decuple result to reduce the round-off error. During the experiments, the temperature was 25–29 °C, and the humidity was 32%–53%.

### 2.6. Verification Test Design

In order to verify the universality of canopy density models, this paper selected different layers with different density which weren’t used in establishing the density model. The actual value was calculated using MATLAB, while the model value was calculated based on the selected canopy density model. The relative errors between model value and actual value were used to analyze the universality of the canopy density model. The verification test was conducted with the same density values and detecting distances with different layers.

## 3. Results and Discussion

### 3.1. Relationship between the Ultrasonic Energy and the Power Supply Voltage

**Figure 6:**
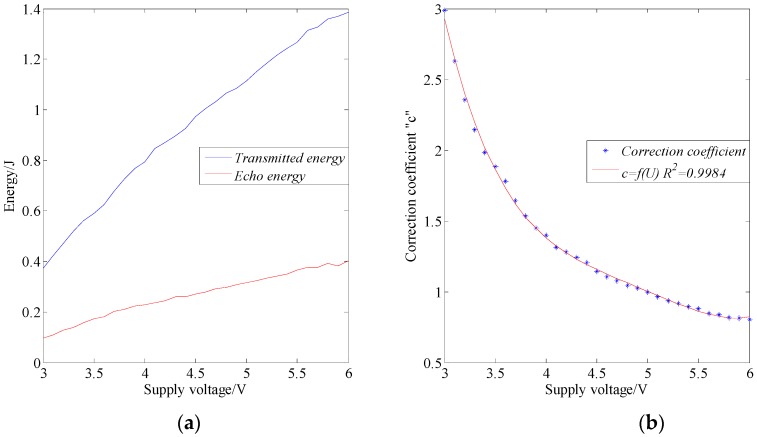
(**a**) Relationship between ultrasonic energy and supply voltage; and (**b**) fitting equation of correction coefficient and supply voltage.

The relationship between the ultrasonic energy and the supply voltage is shown in Figure 6a. It shows that both the transmitted energy and the echo energy went up as the supply voltage increased. Therefore, the stability of supply voltage has an important influence on the time-domain energy analysis. In order to calibrate the transmitted energy, it should be normalized by the correction coefficient. The reference voltage was set as 5.0 V, while the reference transmitted energy was 1.1130 J. The correction coefficient vs. supply voltage curve is shown in Figure 6b. The mathematical equation was obtained as follows, and the value of *R^2^* was 0.9984:

(8) $$c=0.0894U^{4}-1.7704U^{3}+13.1537U^{2}-43.7620U+56.3865,3.0\leq U\leq 6.0$$

where *U* is the supply voltage in V, and *c* is the correction coefficient.

The normalized transmitted energy was multiplied by transmitted energy and correction coefficient, normalized echo energy was echo energy multiplied by echo energy and correction coefficient, and the formula was obtained as follows:

(9) $$E_{N}=c\times E$$

where *E_N_* is the normalized energy, *c* is the correction coefficient, and *E* is the calculation energy.

The normalized echo energy corrected by this coefficient can reduce the deviation caused by supply voltage variation, but it cannot totally eliminate the deviation. Figure 6a shows that the slopes of the transmitted energy variation and the echo energy variation were different, thus the normalized echo energy still has deviation. In order to obtain a uniform reference, it is necessary to ensure the stability of the sensor supply voltage.

### 3.2. Beam Width of the Ultrasonic Sensor

Table 2 shows the result of the beam width experiment. The results showed that the beam width was different at different detection distances. In order to avoid detecting the boundary of the test bench, the detection points were set at 20 cm inside the test bench.

### 3.3. Canopy Density Model

Results of the canopy model experiments with 4 layers are shown in Table 3. The equation coefficients and statistical parameters were calculated (Table 4).

As *F_Lf_* < 1, and *F* > *F*_0.90_(5,5) = 3.45, the flowing model was acceptable. The value of *F*_12_ was less than *F*_0.90_(1,2) = 8.53, so the term *x*_1_*x*_2_ could be ignored. The canopy density model equation with four layers was obtained as follows:

(10) $$10y=2.750+0.376x_{1}-1.262x_{2}-0.533x_{1}^{\prime }+0.434x_{2}^{\prime }$$

(11) $$x_{1}=\frac{z_{1}-620.21}{440.91},x_{2}=\frac{z_{2}-1}{0.43}$$

(12) $$y=-2.742\times 10^{-7}z_{1}^{2}+0.2348z_{2}^{2}+4.225\times 10^{-4}z_{1}-0.7831z_{2}+0.6609$$

where *z*_1_ is the canopy density in g/m^3^, *z*_2_ is the distance in m, and *y* is the echo energy. Similar experiments were conducted to establish canopy density models with three layers (Table 5). The equation coefficients and statistical parameters were calculated (Table 6).

As *F_Lf_* < 1, and *F* > *F*_0.90_(5,5) = 3.45, the model was acceptable. The value of *F*_12_ was less than *F*_0.90_(1,2) = 8.53, so the term *x*_1_*x*_2_ could be ignored. The canopy density model equation with three layers was obtained as follows:

(13) $$10y=2.602+0.735x_{1}-1.207x_{2}-0.28x_{1}^{\prime }+0.29x_{2}^{\prime }$$

(14) $$x_{1}=\frac{z_{1}-620.21}{440.91},x_{2}=\frac{z_{2}-1}{0.43}$$

(15) $$y=-1.440\times 10^{-7}z_{1}^{2}+0.1596z_{2}^{2}+3.454\times 10^{-4}z_{1}-0.5945z_{2}+0.5386$$

where *z*_1_ is the canopy density in g/m^3^, *z*_2_ is the distance in m, and *y* is the echo energy.

### 3.4. Model Equation Selection

With the purpose of selecting a better model equation to simplify the application in practice, experimental data with four layers and three layers were used to contrast the two different model equations. The results are shown in Table 7 and Table 8.

The model echo energy was calculated based on canopy density models for four layers and three layers (Equations (12) and (15)). Table 7 shows that the relative errors of model echo energy and actual normalized echo energy with three layers and four layers were different. The maximum relative error was 53.47%, and the average relative error was 16.07%. The maximum relative error of the model with four layers was 19.57% with the average relative error of 8.80%. Table 8 shows that the maximum relative error of the model with three layers was 26.14%, and the average relative error was 8.26%. The maximum relative error of the model with four layers was 26.83% and the average relative error was 10.76%. More importantly, the variance of relative error for the model with four layers was smaller than the model with three layers in those comparisons. The result of the model equation comparison showed that the canopy density model with four layers was more universal than the canopy density model with three layers, thus this paper selected the canopy density model with four layers as the optimal equation.

### 3.5. Model Equation Verification

The canopy density model universal analysis with five layers of leaves is shown in Table 9. The model value was calculated based on the canopy density model with four layers (Equation (12)). Table 9 shows that the relative errors of the model value and actual normalized echo energy were small. The maximum relative error was 17.68%, the minimum relative error was 1.46% and the average relative error was 8.33%.

Canopy density model universal analysis with four layers is shown in Table 10, which shows that the relative errors of model value and actual normalized echo energy still were small. The maximum relative error was 25.64%, the minimum relative error was 1.23% and the average relative error was 12.61%.

Canopy density model universal analysis with 3 layers is shown in Table 11. The results showed that the relative errors of model value and actual normalized echo energy still were small. 

The maximum relative error was 21.33%, the minimum relative error was 3.31% and the average relative error was 14.19%. Canopy density model universal analysis with two layers is shown in Table 12. The results showed that the relative errors of model value and actual normalized echo energy were acceptable. The maximum relative error was 29.92%, the minimum relative error was 2.32% and the average relative error was 17.98%.

As a consequence of model equation verification, the model equation had a good applicability with different layers, but a higher relative error was experienced with two layers. 

## 4. Conclusions

A method for estimating canopy density of a planar orchard target based on ultrasonic echo energy was studied. Testing indicated that there were strong relationships among the ultrasonic echo energy, detecting distance and canopy density. Two canopy density models with three layers and four layers of leaves were established and compared. The model with four layers was selected as optimal. The verification test results using the optimal model showed that the maximum relative error of model value and actual value with different layers was 17.68%, 25.64%, 21.33% and 29.92%, respectively. The data also suggested the canopy density model with four layers would provide reasonable estimates for different layers. Therefore, it could be used as a control basis in precision sprayers to adjust liquid flow rate and airflow rate.

The relationship between the ultrasonic energy and the power supply voltage showed that the slopes of transmitted energy variation and echo energy variation were different, so normalized echo energy calculated still deviated from the actual echo energy. If supply voltage could be stabilized, the errors can be further reduced without normalization. Future work will focus on field experiments in combination with the real situation of orchard targets.

## Figures and Tables

**Table 1:** Factor levels coding.

Factor	*Z_l__j_*	*Z_u__j_*	*Z*_0_*_j_*	*∆_j_*	*−γ*	−1	0	1	*γ*
Density (*Z*_1_) [g/m^3^]	112.77	1127.66	620.21	440.91	112.77	179.31	620.21	1061.12	1127.66
Distance (*Z*_2_) [m]	0.5	1.5	1.0	0.43	0.5	0.57	1.0	1.43	1.5

**Table 2:** Results of beam width experiments.

Tests	*S* [m]	*W_L_* [cm]	*W_R_* [cm]	Average of *W_L_* [cm]	Average of *W_R_* [cm]
1	0.5	6	7	6.3	6.8
2		6.5	6.5		
3		6.5	7		
4	0.57	8	7	7.7	7.0
5		7.5	7		
6		7.5	7		
7	1.0	11	12	10.5	11.2
8		10.5	11		
9		10	10.5		
10	1.43	12	13	12.0	12.3
11		11	12		
12		13	12		
13	1.5	16	14	15.0	14.2
14		15	14.5		
15		14	14		

**Table 3:** Results of canopy density model experiments with 4 layers of leaves.

*Z*_1_ [g/m^3^] (*x*_1_)	*Z*_1_ [m] (*x*_2_)	*x*_1_*x*_2_	*x*_1_*^’^*	*x*_2_*^’^*	Transmitted Energy [J]	Echo Energy [J]	Normalized Echo Energy [J]	Decuple Normalized Echo Energy [J]
1061.12(1)	1.43(1)	1	0.396	0.396	1.2738	0.1815	0.1586	1.586
1061.12(1)	0.57(−1)	−1	0.396	0.396	1.2601	0.4878	0.4309	4.309
179.31(−1)	1.43(1)	−1	0.396	0.396	1.2601	0.1528	0.1350	1.350
179.31)(−1)	0.57(−1)	1	0.396	0.396	1.3354	0.4230	0.3526	3.526
1127.66(r)	1.0(0)	0	0.716	−0.604	1.3347	0.3338	0.2784	2.784
112.77(−r)	1.0(0)	0	0.716	−0.604	1.3524	0.1818	0.1496	1.496
620.21(0)	1.5(r)	0	−0.604	0.716	1.3291	0.2265	0.1897	1.897
620.21(0)	0.5(−r)	0	−0.604	0.716	1.3036	0.5774	0.4930	4.930
620.21(0)	1.0(0)	0	−0.604	−0.604	1.3211	0.3670	0.3092	3.092
620.21(0)	1.0(0)	0	−0.604	−0.604	1.3249	0.3189	0.2679	2.679
620.21(0)	1.0(0)	0	−0.604	−0.604	1.3363	0.3126	0.2603	2.603

**Table 4:** Equation coefficients and statistical parameters of canopy density model.

Regression Equation Parameters		Test for Lack of Fit of Density Model		Equation Parameter Hypothesis Test	
*b*_0_	2.750	*S_R_*	0.301	*F*_1_	13.601
*b*_1_	0.376	*S_T_*	13.243	*F*_2_	153.131
*b*_2_	−1.262	*S_Lf_*	0.163	*F*_12_	0.037
*b*_12_	−0.137	*S_e_*	0.138	*F*_11_	14.276
*b*_11_	−0.533	*F_Lf_*	0.786	*F*_22_	9.481
*b*_22_	0.434	*f_R_*	5	*F*	43.971
		*F_T_*	5		
		*f_Lf_*	3		
		*f_e_*	2		

**Table 5:** Results of canopy density model experiments with three layers of leaves.

*Z*_1_ [g/m^3^] (*x*_1_)	*Z*_1_ [m] (*x*_2_)	*x*_1_*x*_2_	*x*_1_*^’^*	*x*_2_*^’^*	Transmitted Energy [J]	Echo Energy [J]	Normalized Echo Energy [J]	Decuple Normalized Echo Energy [J]
1061.12(1)	1.43(1)	1	0.396	0.396	1.3381	0.2098	0.1745	1.745
1061.12(1)	0.57(−1)	−1	0.396	0.396	1.3362	0.5665	0.4718	4.718
179.31(−1)	1.43(1)	−1	0.396	0.396	1.3506	0.1185	0.0977	0.977
179.31)(−1)	0.57(−1)	1	0.396	0.396	1.3301	0.3277	0.2742	2.742
1127.66(r)	1.0(0)	0	0.716	−0.604	1.3468	0.3936	0.3253	3.253
112.77(−r)	1.0(0)	0	0.716	−0.604	1.3531	0.1693	0.1393	1.393
620.21(0)	1.5(r)	0	−0.604	0.716	1.3524	0.2002	0.1648	1.648
620.21(0)	0.5(−r)	0	−0.604	0.716	1.3424	0.5427	0.4499	4.499
620.21(0)	1.0(0)	0	−0.604	−0.604	1.3512	0.3146	0.2591	2.591
620.21(0)	1.0(0)	0	−0.604	−0.604	1.3569	0.2879	0.2361	2.361
1061.12(1)	1.43(1)	0	−0.604	−0.604	1.3426	0.3253	0.2697	2.697

**Table 6:** Equation coefficients and statistical parameters of Canopy density model.

Regression Equation Parameters		Test for Lack of Fit of Density Model		Equation Parameter Hypothesis Test	
*b*_0_	2.602	*S_R_*	0.144	*F*_1_	121.882
*b*_1_	0.735	*S_T_*	14.193	*F*_2_	328.565
*b*_2_	−1.207	*S_Lf_*	0.085	*F*_12_	0.182
*b*_12_	−0.302	*S_e_*	0.059	*F*_11_	9.270
*b*_11_	−0.280	*F_Lf_*	0.963	*F*_22_	9.915
*b*_22_	0.290	*f_R_*	5	*F*	95.589
		*f_T_*	5		
		*f_Lf_*	3		
		*f_e_*	2		

**Table 7:** Result of model equation contrast with 4 layers of leaves.

Density [g/m^3^]	Distance [m]	Normalized Echo Energy [J]	Model Equation with Three Layers		Model Equation with Four Layers	
			Calculated Value [J]	Relative Error [%]	Calculated Value [J]	Relative Error [%]
1061.12	1.43	0.1586	0.2099	32.35	0.1896	19.57
1061.12	0.57	0.4309	0.4513	4.74	0.4420	2.59
179.31	1.43	0.1350	0.0628	53.47	0.1168	13.50
179.31	0.57	0.3526	0.3042	13.71	0.3692	4.71
1127.66	1.0	0.2784	0.3036	9.06	0.2606	6.38
112.77	1.0	0.1496	0.1343	10.23	0.1768	18.14
620.21	1.50	0.1897	0.1549	18.33	0.2012	6.08
620.21	0.50	0.4930	0.4356	11.64	0.4947	0.34
620.21	1.0	0.3092	0.2560	17.19	0.2892	6.45
620.21	1.0	0.2679	0.2560	4.43	0.2892	7.96
620.21	1.0	0.2603	0.2560	1.66	0.2892	11.10

**Table 8:** Result of model equation contrast with 3 layers of leaves.

Density [g/m^3^]	Distance [m]	Normalized Echo Energy [J]	Model Equation with Three Layers		Model Equation with Four Layers	
			Calculated Value [J]	Relative Error [%]	Calculated Value [J]	Relative Error [%]
1061.12	1.43	0.1745	0.2192	25.60	0.1608	7.87
1061.12	0.57	0.4718	0.4560	3.37	0.4304	8.78
179.31	1.43	0.0977	0.0721	26.14	0.0882	9.75
179.31	0.57	0.2742	0.3089	12.64	0.3478	26.83
1127.66	1.0	0.3253	0.3101	4.67	0.2504	23.02
112.77	1.0	0.1393	0.1408	1.09	0.1568	12.54
620.21	1.50	0.1648	0.1648	0.01	0.1711	3.85
620.21	0.50	0.4499	0.4401	2.19	0.4846	7.71
620.21	1.0	0.2591	0.2625	1.32	0.2692	3.88
620.21	1.0	0.2361	0.2625	11.18	0.2692	14.00
620.21	1.0	0.2697	0.2625	2.65	0.2692	0.19

**Table 9:** Canopy density model universal analysis with 5 layers of leaves.

Density [g/m^3^]	Distance [m]	Transmitted Energy [J]	Echo Energy [J]	Normalized Echo Energy [J]	Model Value [J]	Relative Error [%]
319.15	0.8	1.3330	0.3631	0.3032	0.3076	1.46
319.15	1.2	1.3330	0.2002	0.1672	0.1902	13.78
478.72	0.8	1.3215	0.3886	0.3273	0.3402	3.92
478.72	1.2	1.3271	0.2540	0.2130	0.2228	4.59
744.68	0.8	1.3087	0.4354	0.3703	0.3634	1.88
744.68	1.2	1.3267	0.2500	0.2098	0.2460	17.26
904.26	0.8	1.3153	0.3995	0.3381	0.3587	6.10
904.26	1.2	1.3285	0.2448	0.2050	0.2413	17.68

**Table 10:** Canopy density model universal analysis with four layers of leaves.

Density [g/m^3^]	Distance [m]	Transmitted Energy [J]	Echo Energy [J]	Normalized Echo Energy [J]	Model Value [J]	Relative Error [%]
319.15	0.8	1.2742	0.3378	0.2951	0.3076	4.26
319.15	1.2	1.3317	0.1985	0.1659	0.1902	14.63
478.72	0.8	1.3245	0.4098	0.3444	0.3402	1.23
478.72	1.2	1.3256	0.2112	0.1773	0.2228	25.64
744.68	0.8	1.3112	0.3698	0.3139	0.3634	15.78
744.68	1.2	1.3274	0.2372	0.1989	0.2460	23.68
904.26	0.8	1.3192	0.3754	0.3168	0.3587	13.24
904.26	1.2	1.3211	0.2936	0.2474	0.2413	2.46

**Table 11:** Canopy density model universal analysis with 3 layers of leaves.

Density [g/m^3^]	Distance [m]	Transmitted Energy [J]	Echo Energy [J]	Normalized Echo Energy [J]	Model Value [J]	Relative Error [%]
319.15	0.8	1.3285	0.3267	0.2737	0.3076	12.41
319.15	1.2	1.3235	0.2340	0.1967	0.1902	3.31
478.72	0.8	1.3184	0.3805	0.3213	0.3402	5.88
478.72	1.2	1.3272	0.2189	0.1836	0.2228	21.33
744.68	0.8	1.3155	0.3546	0.3000	0.3634	21.13
744.68	1.2	1.3260	0.2416	0.2028	0.2460	21.31
904.26	0.8	1.3077	0.3939	0.3352	0.3587	6.99
904.26	1.2	1.3221	0.2365	0.1991	0.2413	21.17

**Table 12:** Canopy density model universal analysis with two layers of leaves.

Density [g/m^3^]	Distance [m]	Transmitted Energy [J]	Echo Energy [J]	Normalized Echo Energy [J]	Model Value [J]	Relative Error [%]
319.15	0.8	1.3148	0.3293	0.2788	0.3076	10.35
319.15	1.2	1.3806	0.2306	0.1859	0.1902	2.32
478.72	0.8	1.3110	0.3378	0.2867	0.3402	18.63
478.72	1.2	1.3852	0.2232	0.1793	0.2228	24.21
744.68	0.8	1.3248	0.3541	0.2975	0.3634	22.15
744.68	1.2	1.3363	0.2343	0.1951	0.2460	26.04
904.26	0.8	1.3202	0.3275	0.2761	0.3587	29.92
904.26	1.2	1.3277	0.2611	0.2188	0.2413	10.26
